# The Multifaceted Role of Aldehyde Dehydrogenases in Prostate Cancer Stem Cells

**DOI:** 10.3390/cancers13184703

**Published:** 2021-09-20

**Authors:** Jakob Püschel, Anna Dubrovska, Ielizaveta Gorodetska

**Affiliations:** 1 OncoRay-National Center for Radiation Research in Oncology, Faculty of Medicine and University Hospital Carl Gustav Carus, Technische Universität Dresden and Helmholtz-Zentrum Dresden-Rossendorf, 01309 Dresden, Germany; J.Puschel@students.uu.nl; 2 National Center for Tumor Diseases (NCT), Partner Site Dresden, German Cancer Research Center (DKFZ), Heidelberg, Faculty of Medicine and University Hospital Carl Gustav Carus, Technische Universität Dresden, and Helmholtz-Zentrum Dresden-Rossendorf (HZDR), 01307 Dresden, Germany; 3 Helmholtz-Zentrum Dresden-Rossendorf, Institute of Radiooncology-OncoRay, 01328 Dresden, Germany; 4 German Cancer Consortium (DKTK), Partner Site Dresden and German Cancer Research Center (DKFZ), 69120 Heidelberg, Germany

**Keywords:** prostate cancer, cancer stem cells, aldehyde dehydrogenase, cancer stem cell-targeted therapy, cancer biomarkers

## Abstract

**Simple Summary:**

Cancer stem cells (CSCs) are an engine of tumor progression and a source of tumor therapy resistance and regrowth after treatment. Modern conventional therapies can eliminate most non-CSCs, while CSCs often survive cancer treatment, leading to tumor relapse and metastases. Prostate cancer (PCa) is a disease that affects 1 in 8 men in their lifetime. Although the 5-year survival rate of patients with localized or regional PCa is close to 100%, it dramatically decreases to 30% for the patients with distant metastases. Thus, targeting CSCs might be a promising approach to overcome tumor resistance and increase the efficiency of the current cancer treatment strategies. A high aldehyde dehydrogenase (ALDH) activity is a widely accepted marker of prostate CSCs. This review discusses the current state of research regarding the role of individual ALDH enzymatic proteins in PCa development and progression, their possible therapeutic targeting, and future development in this field.

**Abstract:**

Cancer stem cells (CSCs) are the only tumor cells possessing self-renewal and differentiation properties, making them an engine of tumor progression and a source of tumor regrowth after treatment. Conventional therapies eliminate most non-CSCs, while CSCs often remain radiation and drug resistant, leading to tumor relapse and metastases. Thus, targeting CSCs might be a powerful tool to overcome tumor resistance and increase the efficiency of current cancer treatment strategies. The identification and isolation of the CSC population based on its high aldehyde dehydrogenase activity (ALDH) is widely accepted for prostate cancer (PCa) and many other solid tumors. In PCa, several ALDH genes contribute to the ALDH activity, which can be measured in the enzymatic assay by converting 4, 4-difluoro-4-bora-3a, 4a-diaza-s-indacene (BODIPY) aminoacetaldehyde (BAAA) into the fluorescent product BODIPY-aminoacetate (BAA). Although each ALDH isoform plays an individual role in PCa biology, their mutual functional interplay also contributes to PCa progression. Thus, ALDH proteins are markers and functional regulators of CSC properties, representing an attractive target for cancer treatment. In this review, we discuss the current state of research regarding the role of individual ALDH isoforms in PCa development and progression, their possible therapeutic targeting, and provide an outlook for the future advances in this field.

## 1. Introduction

Prostate cancer (PCa) is a disease that will prognostically affect 1 in 8 men in their lifetime. The American Cancer Society indicates 5-year survival rates of 100% for localized or regional PCa. This survival rate plummets to 30% for the patients with distant metastases [1]. Androgens play an essential role in PCa development and progression by facilitating oncogenic transcriptional programs [2]. Therefore, androgen deprivation therapy (ADT) is an important component of the PCa treatment strategy [3,4]. However, most PCa patients showing an initial response to ADT or surgical castration eventually develop castration-resistant prostate cancer (CRPC) with or without the metastases [5]. Despite a number of novel treatment approaches (e.g., novel antiandrogens, poly(adenosine diphosphate [ADP]-ribose) polymerase (PARP) inhibitors, immune-directed or radioligand therapy), CRPC is considered incurable [5,6]. Therefore, there is a high necessity for novel therapeutic strategies to improve a patient’s outcome for metastatic and castration-resistant disease.

One of the factors hampering the treatment success is attributed to cancer stem cells (CSC). First described and characterized by Bonnet and Dick in acute myeloid leukemia, the population of CSCs was found in solid tumors, including prostate cancer [7,8]. CSCs exhibit unique features, such as self-renewal, differentiation, and the capability to withstand radio- or chemotherapy [9,10]. Therefore, targeting the population of CSCs is crucial for therapy success and opens new ways to improve the patient’s outcomes.

**Figure 1:**
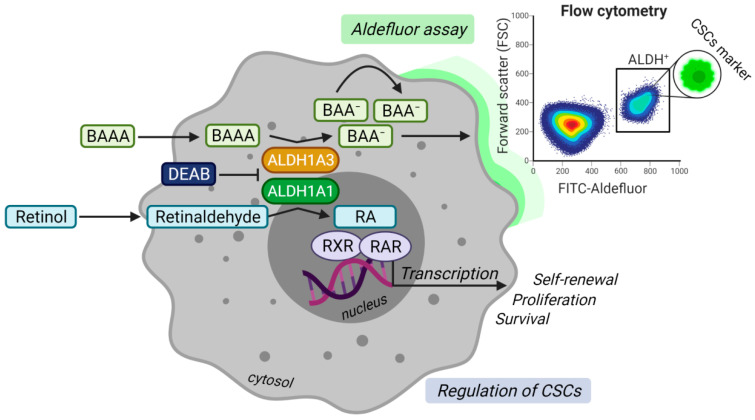
Model illustrating the enzymatic function of aldehyde dehydrogenases (ALDH)1A1 and ALDH1A3 proteins in ALDEFLUOR assay and the regulation of cancer stem cells (CSCs) through retinoic acid (RA) signaling. In the upper panel, prostate cancer cells expressing ALDH1A1 and ALDH1A3 convert uncharged fluorescent ALDH substrate BODIPY-aminoacetaldehyde (BAAA) into negatively charged BODIPY-aminoacetate (BAA^−^). BAA^−^is retained inside the cell, making the subset of ALDH+ cells highly fluorescent. Diethylaminobenzaldehyde (DEAB), a specific ALDH inhibitor, prevents the cells from becoming fluorescent and is used as a negative control. The fluorescence can be measured by flow cytometry. The lower panel demonstrates that ALDH1A1 and ALDH1A3 proteins regulate CSCs via the production of retinoic acid (RA) from retinaldehyde (also known as retinal). RA binds to heterodimers formed by retinoid x receptor (RXR) and retinoic acid receptor (RAR) in the nucleus. Activation of the receptors complex stimulates transcription of the target genes regulating self-renewal, proliferation, and survival. Created with BioRender.

A high activity of aldehyde dehydrogenases (ALDH) measured by ALDEFLUOR assay is one of the most widely described stem cell markers for PCa [11,12]. The assay evaluates the enzymatic production of a fluorescent negatively charged 4, 4-difluoro-4-bora-3a, 4a-diaza-s-indacene (BODIPY)-aminoacetate (BAA) converted by ALDH enzymes from an uncharged BODIPY-aminoacetaldehyde (BAAA), as shown in Figure 1 [13]. To date, the family of human ALDH isogenes comprises 19 members [14]. ALDHs catalyze the irreversible reaction of aldehydes to corresponding carboxylic acids, subsequently protecting cancer cells from the reactive oxygen species (ROS) generated, e.g., after radiotherapy and some types of chemotherapies [14,15].

In PCa, seven ALDH genes (ALDH1A1, ALDH1A3, ALDH3A1, ALDH4A1, ALDH7A1, ALDH9A1, and ALDH18A1) are found elevated over healthy prostate tissues and potentially contribute to the ALDH activity measured by ALDEFLUOR assay. Although each ALDH isoform plays an individual role in cancer progression, the studies suggest a mutual interplay between many of them [13,16,17]. ALDH1A1 and ALDH1A3 are the most investigated genes contributing to ALDEFLUOR activity in PCa and considered pivotal isoforms due to their involvement in retinoic acid (RA) signaling (Figure 1). While some isoforms are established as markers for various tumor entities, many ALDH isozymes stay unnoticed in their contribution to, e.g., an altered metabolism that is one of the hallmarks of cancer [18,19]. Hence, it is crucial to understand the individual role of every isoform gene in the onset, metastatic spread, relapse, and metabolic deregulation of PCa (Table 1). 

**Figure 2:**
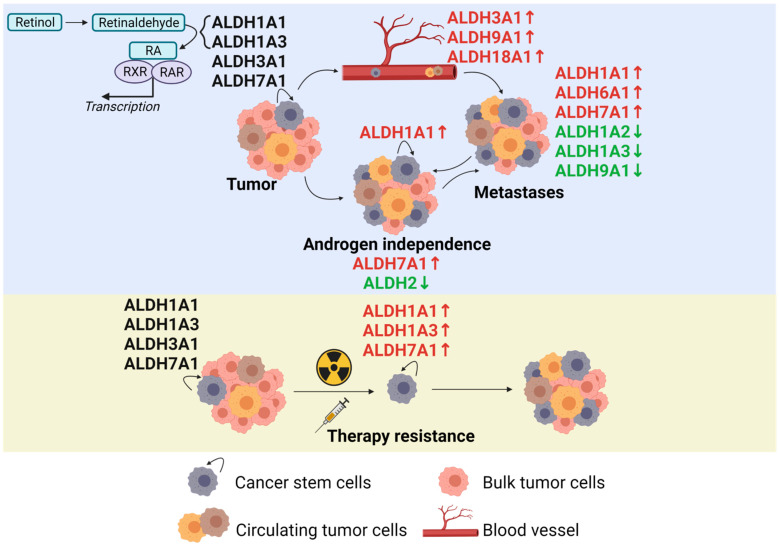
The role of the ALDH proteins in prostate cancer development, metastatic spread, and therapy resistance according to the current state of the literature. Created with BioRender.

This review compiles the current state of research regarding the individual ALDH isoform genes and their contribution to PCa development and progression to outline the potential therapy targets and elicit deregulated expression patterns upon malignancy. We review the individual role of every isogene in the context of PCa according to the current state of the literature (Figure 2). We also deliberate the preclinical development of ALDH-targeted therapies and summarize the clinical potential of these agents. Finally, we discuss the milestones in the research regarding the ALDH gene family in PCa and give an outlook for the future developments in this field.

## 2. ALDH1A1

ALDH1A1 represents the most investigated isoform of all of the ALDH genes. Together with ALDH1A2, ALDH1A3, and ALDH8A1, it belongs to the retinal metabolizing enzymes, contributing to the synthesis of retinoic acid. A high activity of aldehyde dehydrogenases is a feature of normal and cancer stem cells that allows the detoxification of reactive oxygen species, specifically aldehydes, and can be measured by ALDEFLUOR assay. ALDH^+^ cancer cells also possess abilities for self-renewal and differentiation. ALDH1A1 and ALDH1A3 were recognized as the main contributors to ALDEFLUOR enzymatic activity in prostate cancer cells (Figure 1) [23,42,43]. ALDH1A1 is a marker for normal and cancer stem cells of various tissue types, including a healthy and a cancerous prostate [44,45,46,47]. Li and co-authors found that ALDH1A1^+^ cells make only a small subset of CD44^+^ cell population in a basal component of normal prostate tissues. This study also demonstrated that ALDH1A1 highly expresses in prostate cancer tissues, including secretory epithelial cells and neuroendocrine cells [47]. Similar results were obtained in a tissue microarray study on Iranian patients, indicating an increased ALDH1A1 expression in PCa over prostatic intraepithelial neoplasia (PIN) and benign prostatic hyperplasia (BPH) samples. The authors also found that ALDH1A1 expressing cells appeared more frequently in samples with higher prostate-specific antigen (PSA) levels and a higher Gleason score [48].

Oppositely, two studies showed that ALDH1A1 expression is not significantly elevated between primary PCa samples, BPH, and a healthy prostate [49,50]. Nevertheless, the study of Le Magnen et al. also showed that within the PCa samples, higher ALDH1A1 expression correlated with a higher Gleason score and the percentage of ALDH^+^ cells. Elevated ALDH1A1 levels adversely affected the overall survival in hormone-naїve patients and were observed more frequently in CRPC than in low-stage cancer. However, high ALDH1A1 expression does not predict worse overall survival in castration-resistant PCa [45].

A study by Nastały et al. comprising patient samples of primary PCa, hormone naїve lymph node (LN) metastases, castration-resistant visceral or bone metastases (*n* = 551) revealed that a high ALDH1A1 expression correlated with a higher T-stage and Gleason score, as well as a lower time to biochemical recurrence [21]. In contrast, high ALDH1A1 expression in the intratumoral adjacent stromal tissues was associated with a longer time to biochemical recurrence, lower T-stage, Gleason score, and N0-stage [21]. Interestingly, these results were in line with a study reporting similar findings in stromal cells of triple-negative breast cancer patient samples, where higher expression led to better disease-free and overall survival [51]. Of note, the expression of ALDH1A1 in prostate tumor tissues was associated with basal cytokeratin CK14. In contrast, ALDH1A1 expression in stromal cells was frequently observed in tumors with high levels of luminal cytokeratins CK8/18 and rarely found in tumor specimens with epithelial-mesenchymal transition (EMT) phenotype. Furthermore, ALDH1A1 expression was found more frequently in metastatic tumors than in primary tumor tissues, whereas stromal ALDH1A1 expression appeared more frequently in primary tumors than in metastatic tumors [21]. These data suggest that ALDH1A1 positive tumor stroma might impede tumor growth, whereas ALDH1A1 expression in cancer cells promotes tumor progression. 

The ALDH1A1 expression levels were elevated in the population of ALDH^+^ cells with high ALDEFLUOR activity. The genetic knockdown of ALDH1A1 expression or chemical inhibition of ALDH1A1 activity resulted in the downregulation of ALDEFLUOR positive cell populations [52,53]. On the other hand, the inhibition of ALDH activity with a non-isoform specific inhibitor DEAB reduced spherogenicity in a PCa cell line-dependent manner [54]. The ALDEFLUOR activity is used in combination with other putative stem cell markers to more precisely define prostate CSC populations. For example, Qin et al. used a combination of ALDH^+^/CD44^+^/α2β1^+^ to identify highly tumorigenic castration-resistant CSCs in the basal PSA^−/lo^ subset of prostate tumors [55]. On the other hand, ALDH1A1 is mutually exclusive with some other putative prostate CSC markers, such as side population cells. The side population assay quantifies the efflux of fluorescent Hoechst33342 dye via the ABC transporter family, thereby giving a measure of cells’ capability to efflux cytotoxins. ALDH1A1 and ABCG2 (another marker for progenitor cells) appeared mutually exclusive in side population assay as the ALDH1A1 expression was enriched in a non-side population [54].

A high ALDH1A1 expression indicates increased tumor resistance to chemo- and radiotherapy. A combination of ALDH1A1 inhibition with the conventional treatment is a promising strategy for developing more efficient cancer therapies. In particular, ALDH1A1 expression and activity can be inhibited by retinoic acid—a product of an ALDH1A1-driven enzymatic reaction [56,57,58]. Treatment with all-trans-retinoic acid (ATRA) was the first differentiation therapy being used for patients with acute promyelocytic leukemia (APL) for over 60 years [59]. The mechanisms of ATRA action are based on the reactivation of gene transcription repressed by PML–RARA protein present in all APL patients. This protein is a chimeric gene product caused by a reciprocal t(15;17) translocation. PML-RARA prevents granulocyte differentiation by binding to the retinoic acid receptor responsive elements in the promoters of genes responsible for this differentiation program and recruiting the co-repressor proteins and epigenetic modifiers. The treatment of APL with ATRA leads to the dissociation of these repressor complexes and triggers the gene expression of the RARA-responsive genes [59]. The ATRA treatment was shown to sensitize different tumor types to the conventional treatment [56,58,60,61] and inhibit CSC populations [57,58,62]. In PCa, ATRA was shown to inhibit the proliferation of AR-negative cells by activating cyclin-dependent kinase 5 (Cdk5) and p27 expression [63]. The ATRA treatment also upregulated homeobox HOXB13 protein expression through decreasing the levels of enhancer of zeste 2 polycomb repressive complex 2 subunit (EZH2) and (DNA methyltransferase 3 beta) (DNMT3b), which form a repression complex on the *HOXB13* gene promoter [64]. Furthermore, ATRA-induced HOXB13 expression in NEPC cells converted them to less aggressive and more treatable prostate adenocarcinoma [65]. 

The ALDH activity and ALDH1A1 expression were associated with a PCa response to radiotherapy. PCa cells with increased ALDH activity displayed enhanced radioresistance [23,53,66]. Cojoc and co-workers demonstrated a direct regulation of ALDH1A1 transcription by the β-catenin-TCF complex. The chemical inhibition of the WNT/β-catenin signaling pathway or the siRNA-mediated knockdown of β-catenin led to radiosensitization and a decreased ALDH1A1 expression [66]. ALDH1A1 mRNA expression is increased in radioresistant DU145 PCa cells compared to their more radiosensitive parental counterparts [23]. A H3K36me3 methylation mark of the ALDH1A1 promoter was found in response to irradiation with 4Gy of X-rays and was associated with an increase in ALDH1A1 transcription, indicating tumor cell reprogramming. When the methylation event was inhibited with the global methyltransferase inhibitor DZNeP, a decreased ALDH1A1 and methyltransferase EZH2 expression was observed in parental and radioresistant cell lines [53]. The study suggested the cooperation of EZH2 and BRCA1 in regulating ALDH1A1 expression, PCa stem cells, and tumor radioresistance, and the genetic silencing of EZH2 alone or in combination with BRCA1 knockdown decreased the ALDH1A1 level [52,66]. In support of this finding, Nolan et al. found a connection of ALDH1A1 to heat shock protein 90 (Hsp90) and EZH2. Extracellular Hsp90 increased the ALDH1A1 activity together with other markers of stemness. The inhibition of Snail or EZH2 decreased the ALDH1A1 activity and provided further evidence for the epigenetic regulation of ALDH1A1 by EZH2 [67]. 

The immunohistochemical staining of various PCa cell lines and primary PCa epithelial tissues revealed a primarily cytoplasmic expression and some nuclear localization of ALDH1A1 [50]. This study showed that the chemical inhibition of ALDH1A1 by different imidazo[1,2-α]pyridine derivatives led to the decreased proliferation and clonogenicity in a panel of established PCa cell lines and patient-derived primary epithelial cells [50]. 

Silybin is a mixture of compounds found in milk thistle seeds and exerting a high anticancer effect on prostate tumor cells. Jiang and co-authors discovered that this effect might be explained by silybin’s negative effect on ALDH1A1 expression. Further, the expression of retinoic acid receptor β and Ets1 (downstream targets of the retinoic acid signaling pathway) were downregulated as well. This study showed that cells with a high expression of ALDH1A1 treated with silybin displayed decreased invasion, migration, and proliferation [68]. The ALDH1A1 expression was reduced upon the treatment of the prostate stromal myofibroblast cell line WPMY-1 (derived from the same donor as prostate epithelial RWPE-1 cell line) with TGF-β or after silencing of Dickkopf-3 (Dkk3). However, Dkk3 silencing in RWPE-1 cells did not have any effect on ALDH1A1 expression [69]. The ALDH1A1 was upregulated in the enzalutamide-resistant subline of the C4-2 cell line. However, the knockdown of β-catenin with or without additional enzalutamide treatment decreased the ALDH1A1 expression and spheroid formation with a more pronounced effect from the combination treatment [70]. Lastly, the inhibition of androgen receptor (AR)-dependent transcriptional program by enzalutamide resulted in a decreased ALDH1A1 expression in the androgen-sensitive LNCaP cell line but not androgen-independent C4-2B, hinting at a transcriptional regulation of ALDH1A1 depending on the AR status of the cell line [23].

Taken together, ALDH1A1 proves to be a marker for prostate CSCs, and its expression increases with disease progression. However, conflicting studies exist regarding its differential expression before and after the onset of PCa. Multiple studies have shown an interconnection with methyltransferase EZH2 and the WNT/β-catenin signaling pathway as well as the transcriptional regulation by AR. The targeting of either pathway may represent a way to attenuate ALDH1A1 expression and target the CSC populations. 

## 3. ALDH1A2

ALDH1A2 is widely recognized as a tumor suppressor gene in prostate cancer. Together with ALDH1A1 and ALDH1A3, ALDH1A2 is one of the main enzymes that catalyze the oxidation of all-trans retinaldehyde to all-trans retinoic acid. PCa and a normal prostate contain similar retinol levels, but a normal prostate displays much lower levels of retinoic acid (the main product of the reaction catalyzed by ALDH1A2). The low expression of ALDH1A2 in PCa might partially explain this effect [71]. An immunohistochemical analysis of ALDH1A2 expression displayed low cytoplasmic staining in human prostate cancer paraffin sections relative to normal human prostate tissues, presumably due to its promoter hypermethylation in PCa samples [16]. Furthermore, ALDH1A2 expression negatively correlated with a recurrence-free survival. Contrarily, the induced expression of ALDH1A2 reduced tumor cell clonogenicity [16]. High levels of ALDH1A2 protein in the cytoplasm and nucleus were observed in non-transgenic murine prostate paraffin sections. In contrast, transgenic adenocarcinoma of the mouse prostate (TRAMP) mice displayed a weak cytosolic and no nuclear staining. These experiments demonstrated that the abrogation of ALDH1A2 expression happens at an early stage of PCa progression and underlines the significance of the retinoid signaling in PCa prevention [72].

The study of Merrick and colleagues modeled the changes of ALDH1A2 expression during the progression from a healthy to malignant prostate. The comparison of the ALDH1A2 transcript level in non-tumorigenic RWPE1 cells versus a malignant subline CAsE-PE (generated by long-term exposure to arsenite) revealed its more than 300-fold downregulation in neoplastic cells [73]. In addition, similarly, decreased ALDH1A2 transcript levels were shown to be part of a signature of genes able to predict the metastatic lethal outcome of patients that underwent radical prostatectomy [74]. In the TCGA-PRAD cohort, a signature of eight ALDH genes (including ALDH1A1, ALDH1A2, ALDH1B1, ALDH2, ALDH3A2, ALDH7A1, ALDH8A1, and ALDH9A1) involved in tryptophan metabolism was predominantly downregulated compared to normal patients, with the fold change of ALDH1A2 being the most severe among the panel [75].

Various possibilities for the regulation of ALDH1A2 in PCa have been suggested. For example, miRNA-186-5p, harboring oncogenic activity, directly targeted ALDH1A2 in RWPE1 cells resulting in its downregulation [76]. The upregulation of transcription factor TBX1 diminished retinoic acid signaling by reducing ALDH1A2 expression [77,78]. Guo and co-authors compiled a competitive endogenous RNA network comprising long non-coding RNAs (lncRNA), miRNAs, and transcripts of selected genes. The signature of these RNAs precisely distinguished between the primary PCa samples and the healthy tissues in a patients’ cohort. The authors identified an interaction of ALDH1A2 with lncRNA RP11-166D19.1, miRNAs hsa-miR-222-3p, and hsa-miR-221-5p; however, the exact nature of such interactions remains yet to be elucidated [79]. 

Three single nucleotide polymorphisms (SNP) of ALDH1A2 were significantly associated with a more prolonged PCa survival but not the diagnosis of prostate cancer. The authors suggested that these SNPs might enhance the ALDH1A2 activity and increase the conversion of retinal, although PCa is usually associated with lower levels of retinoic acid [80]. With the help of the Ingenuity Pathway Analysis Database, BIOGRID 3.4, and STRING 10 databases, Nim et al. derived a signature of two genes to distinguish for shorter and longer disease-free survival in PCa patients: cytochrome P450 26A1 (CYP26A1) and retinol dehydrogenase 10 (RDH10). The altered expression of both genes significantly correlated with a shorter disease-free survival [81]. CYP26A1 is the retinoic acid-metabolizing enzyme highly expressed in several cancers, leading to retinoic acid clearance [82,83]. The RDH gene family displayed diminished expression levels in the tumor specimens [84,85]. The quick metabolism of retinoic acid via elevated CYP26A1 expression and the decreased possibility to synthesize retinal due to decreased RDH levels can explain the plummeting retinoic acid levels observed in the PCa specimens beyond decreased ALDH1A2 expression [71]. Consequently, less retinoic acid is available to induce retinoic acid receptor signaling that was shown to promote differentiation, inhibit proliferation, or induce apoptosis in DU145 cells via the activation of CDK5 [86] or the upregulation of the HOXB13 protein expression, as described earlier [64].

Although ALDH1A1, ALDH1A3, and ALDH8A1 can also synthesize retinoic acid, it is also important to acknowledge that only a decreased level of ALDH1A2 expression has been consistently observed in PCa tissues. Thus, despite the first promising attempts to understand the mechanism underlying the downregulation of ALDH1A2 and its consequences for PCa progression were made, future studies are needed to unravel the importance of this isoform for PCa biology.

## 4. ALDH1A3

The last isoform of the ALDH1A subfamily is the retinaldehyde catabolizing isoform ALDH1A3, which contributes to the ALDH activity measured by ALDEFLUOR assay. The previous study revealed ALDH1A3 as the only ALDH gene expressed higher in ALDH^+^ compared to ALDH^-^ counterparts [52], and the knockdown of ALDH1A3 expression significantly decreased the ALDH^+^ population in a panel of PCa cell lines [23]. Consistent with this finding, the genetic inhibition of ALDH1A3 significantly decreased the stem cell phenotype measured by sphere-forming assay and in vitro radioresistance in PCa cells lines analyzed by radiobiological colony-forming assay [23]. The siRNA-mediated knockdown of ALDH1A3 expression in PCa cells significantly increased the number of residual DNA double-strand breaks after radiotherapy, suggesting an essential role of ALDH1A3 in the regulation of DNA damage repair [53]. The immunohistochemical staining of primary prostate cancer tissues showed that ALDH1A3 expression is confined to luminal cells, while its RNA levels positively correlate with luminal markers and negatively correlate with basal markers [87,88]. Consistent with this finding, ALDH1A3 was identified as one of the AR transcription targets [89]. Furthermore, an RNAseq analysis in a panel of 37 TCGA cohorts revealed that ALDH1A3 was expressed the highest in PCa among all malignancies [88]. 

Various studies observed a higher protein expression of ALDH1A3 in PCa samples compared to BPH samples or non-malignant prostate specimens [49,50,90,91]. Quattrini et al. further demonstrated that the ALDH1A3 protein was expressed the highest among a panel of ALDH isoform in both BPH and PCa samples [50]. However, PCa tissues exhibited a higher ALDH1A3 promoter methylation frequency in comparison to matched healthy prostate counterparts. This finding was also made for the other investigated cancer entities, such as breast, colon, and lung, indicating a rather universal status [92]. These data support the findings showing that low ALDH1A3 expression is associated with worse biochemical recurrence-free survival and shorter progression time to castration resistance [23,24]. Potential explanations for such discrepancy might be: (i) elevated ALDH1A3 expression is crucial for the onset of PCa, but continuously decreases following promoter methylation throughout the progression or (ii) the luminal and non-luminal cells are not separated for quantitative RNA or the promoter methylation analysis. Consequently, high or low expression and promoter methylation could be affected by the ratio of luminal to non-luminal cells. This suggests that only one PCa cell line of a single cell type is insufficient to model the importance of ALDH1A3 as a biomarker in PCa progression but employing multiple cell models, including patient-derived tissue cultures, would be more informative. 

Ali and colleagues investigated the expression of ALDH1A3 between African American men and Caucasian American men, and thereby provided a third potential explanation. The authors reported increased ALDH1A3 expression in the PCa specimens and cell lines of African American descent over the tissues or cell lines of Caucasian American lineage. Regarding the controversial level of ALDH1A3 expression between the healthy and malignant tissues, they showed that the expression was decreased for PCa cell lines of Caucasian descent compared to the benign (Caucasian) RWPE1 cell line. Contrarily, ALDH1A3 expression increased in the malignant cell lines of African American donors in comparison to the benign cell line of African American descent. While this study omits the potential inherent differences of the ALDH1A3 levels depending on the cell type or metastatic site, it is worth acknowledging that racial background can play an important role in the context of ALDH1A3 [93]. This finding might be of a special interest taking into account that African American men suffer from higher mortality rates of PCa [94].

Several studies found that a low ALDH1A3 expression correlated with worse progression-free survival [23,87,88]. Wang and co-authors showed that the knockout of ALDH1A3 expression decreased in vitro invasive capacity of PC3 cells and in vivo xenograft tumor growth [88]. Contrary to this finding, the stable downregulation of ALDH1A3 expression was associated with an increased in vivo metastasizing potential of PCa cells [23,88]. ALDH1A3 is transcriptionally regulated by the AR in androgen-sensitive PCa cells, such as LNCaP [89]. Treatment with dihydrotestosterone (DHT) increased the ALDH1A3 activity and its expression in a dose-dependent manner. This effect was abolished by the inhibition of AR by bicalutamide or the siRNA-mediated knockdown [89]. In a different study, the inhibition of the AR by enzalutamide yielded the same effects on ALDH1A3 gene expression as bicalutamide [23]. Lastly, the authors found an increase in CYP26A1 (a protein that catalyzes the oxidation of retinoic acid to 4-oxo-retinoic acid and is involved in retinoic acid degradation) expression upon the treatment with DHT and all-trans-retinal (the substrate for ALDH1A3) [89].

It has previously been mentioned that ALDH1A1 is directly and positively transcriptionally regulated by the β-catenin/TCF-complex [66]. However, the knockdown or chemical inhibition of β-catenin increased the expression of ALDH1A3 in AR-positive cell lines LNCaP and C4-2B but not AR-negative cell line PC3 [23]. This finding indicates that β-catenin and AR may cooperate in the transcriptional regulation of both ALDH isoforms in a cell line-dependent manner and that the transcription is shifted from ALDH1A1 to ALDH1A3 depending on the level of β-catenin or AR signaling activation, thereby reflecting the dynamic regulation of ALDHs during PCa progression [23]. Another study showed that the knockdown of BRCA1, but not BRCA2, led to an increase in ALDH1A3 expression. Combining BRCA1 knockdown with the genetic silencing of methyltransferase EZH2 decreased or did not change ALDH1A3 gene expression in PCa cell lines but increased the ALDH^+^ populations in these cells, suggesting changes in other isoforms’ expression or posttranslational modifications leading to higher enzymatic activity [52]. The ALDH1A3 knockdown led to radiosensitization and a decreased spherogenicity in various PCa cell lines [23,53]. Wang and co-workers discovered the activation of the PI3K/AKT pathway of LNCaP and VCaP cell lines in response to the ALDH1A3 knockdown [24].

The tumor suppressor miR-187 significantly and inversely correlated with ALDH1A3 and negatively regulated its expression in a set of PCa cell lines [95]. Federer-Gsponer et al. reported mutual exclusivity of the isoforms ALDH1A1 and ALDH1A3 based on the unsupervised clustering of gene expression data in hormone-naїve and CRPC tissue specimens. Notably, a phenotypic cluster associated with aggressive neuroendocrine prostate cancer (NEPC) was derived from the samples with a high ALDH1A1 and low ALDH1A3 expression [87]. Similar results were obtained by Gorodetska and colleagues who analyzed the TCGA-PRAD dataset for the expression of both isoforms. A further validation analysis unraveled an interconnection of ALDH1A1 and ALDH1A3 genes [23]. These findings are of importance for the understanding of how both isoforms contribute to PCa progression and metastases.

The SNP rs4646653 of the ALDH1A3 gene was significantly associated with the risk for PCa [90]. A previously described family of imidazo[1,2-α] pyridine derivatives inhibited the activity of ALDH1A1 and ALDH1A3. In a study on various PCa cell lines, the inhibitors significantly decreased the clonogenicity in a PCa cell line but also in benign and normal prostate cell lines. While this class of inhibitors still falls short of selectivity for malignant tissues, it represents an outstanding possibility to target late PCa stages associated with a high ALDH1 gene expression [50,95,96].

In conclusion, ALDH1A3 contributes to ALDH activity, is involved in conferring radioresistance, spherogenicity, and ALDH activity indicative for stem cell-like populations, and therefore might serve as a marker for prostate CSCs. Generally, high levels of ALDH1A3 were associated with more favorable patient outcomes. Much debate occurs about the varying expression levels of ALDH1A3 between healthy, benign, and malignant tissues. However, various studies have indicated that different criteria, such as cell type, racial background, or AR status, should be considered in interpreting such results. Studies also demonstrated the transcriptional regulation of ALDH1A3 by the AR and presumably by β-catenin, as well as the interconnection with the expression of ALDH1A1. Further studies are needed to validate the molecular mechanisms of ALDH1A3 regulation and find a selectively druggable approach for its targeting.

## 5. ALDH1B1

Not much is known yet about the role of this isoform for PCa pathophysiology. The expression of this isogene was increased in PCa samples over BPH samples [25,50]. The expression levels of ALDH1B1 can be influenced by curcumin, a natural product of turmeric. Curcumin treatment induced apoptosis and increased ROS levels, which could at least partially be explained by decreased levels of ALDH1B1 [97].

## 6. ALDH1L1

ALDH1L1 is involved in the apoptosis of prostate cancer cells, as shown by Ghose et al. [98]. The authors demonstrated that the induction of catalytically active ALDH1L1 expression decreased proliferation and induced apoptosis in the p53-deficient PC3 cell line. The researchers found reduced caspase 8 activity and showed that apoptosis was mediated via mitogen-activated protein kinase (MAPK). In turn, extracellular signal-regulated kinase (ERK) induced Elk1 and c-Jun phosphorylation through c-Jun N-terminal kinases (JNK2). In a follow-up study, the same group proposed another downstream target of JNK1/2 as Bid. JNK1/2-dependent phosphorylation prevents caspase 8-dependent Bid cleavage. Both cleaved and full-length Bid can be translocated into the mitochondria as a part of the apoptotic induction, although the full-length Bid was described as a weaker apoptosis inducer. ALDH1L1 activates JNK1 and JNK2 and increases the level of a full-length Bid in cancer cells as a pro-apoptotic response [99]. Taken together, ALDH1L1 is involved in the controlled mediation of apoptosis in prostate cancer cells. Alternative mRNA splicing of ALDH1L1 seem to be of clinical relevance as two ALDH1L1 variants were found to be expressed higher in either cancer or normal tissues [100]. While plenty of single nucleotide polymorphisms were found for ALDH1L1, none of them were significantly associated with an elevated risk of PCa [101]. The downregulation of DNA methylations of ALDH1L1 was observed more frequently in malignant disease rather than adenoma [102]. The genetic silencing of transcription factor FOXO4 increased the invasion and expression of ALDH1L1, linking it with metastases. FOXO4 was also shown to be inactivated by the PI3K/AKT signaling pathway. The lymph node metastases that occurred in vivo upon FOXO4 knockdown featured higher transcript levels of ALDH1L1, indicating the regulation of ALDH1L1 by the PI3K/AKT signaling pathway [103]. Taken together, ALDH1L1 may be an isoform that is involved in the metastatic spread of PCa.

## 7. ALDH1L2

There is little knowledge about the importance of ALDH1L2 in PCa. Still, it may have implications in cells’ response to radiotherapy as its expression and copy number were increased in radioresistant DU145 cells compared to the parental counterparts [104].

## 8. ALDH2

Isoform ALDH2 is best known for its turnover of acetaldehyde in alcohol metabolism [105]. Conflicting results have been yielded regarding how ALDH2 expression is associated with PCa development and progression. Kim and colleagues observed a decreased expression of ALDH2 in metastatic samples over primary PCa and healthy prostate tissues [106]. This observation is supported by other findings that ALDH2 expression was decreased in patients with the lethal outcome of PCa over patients that survived for at least eight years without metastases after an adjustment for the Gleason score [107]. Multiple other datasets support these findings, as ALDH2 was downregulated in CRPC samples over primary PCa and also diminished in samples of recurrent PCa over non-recurrent tumor tissues. Finally, ALDH2 expression did not correlate with the tumor volume; however, it was described as a part of a gene signature that can be used to predict the visibility of PCa on magnetic resonance imaging (MRI) scans and the biochemical recurrence-free survival [108]. This also applies to ALDH2 expression alone as its higher expression correlated with a better recurrence-free survival and discriminated from CRPC. Furthermore, its elevated expression was shown as part of a six gene prognostic signature for overall and metastasis-free survival [29]. All these findings hint at a tumor-suppressive function of ALDH2. 

However, a study by Ummanni and co-authors found an increased ALDH2 protein expression in PCa compared to the BPH samples [109]. These results were supported by the study of Quattrini et al. on the ALDH2 transcript level [50]. Therefore, a possible explanation could be that the absence of ALDH2 plays an essential role in the pathophysiology of BPH, and subsequently, lower ALDH2 should be found in BPH compared to both PCa and healthy prostate samples. Further research using large prospective patient cohorts is warranted to validate these findings. Furthermore, a particular association of ALDH2 with stem-like prostate cancer cells was suggested by a study of Liu et al. showing increased levels of ALDH2 transcripts in CD44^+^ DU145 cells [110].

Flavopiridol was demonstrated to induce apoptosis in tumor cells via the induction of mitochondrial lesions. The DU145-flavopiridol-resistant cell line had an upregulated ALDH2 expression compared to parental DU145. This might be potentially explained by the protective role of ALDH2 as it is localized in the mitochondria, one of the major sites of ROS production, and ROS levels were significantly elevated in the flavopiridol-resistant cells [111].

Interestingly, ALDH2 seems to represent a tumor suppressor in multiple cancer entities as it was found to be universally downregulated in a panel of 12 different TCGA cohorts [75]. Taken together, ALDH2 has been suggested as a tumor suppressor gene in PCa but can be found in BPH in even lower levels. It may be involved in the response to long-term exposure to chemicals inducing apoptosis and mitochondrial stress. The mechanism by which ALDH2 is being downregulated upon malignant onset remains to be elucidated.

## 9. ALDH3A1

ALDH3A1 was widely investigated in prostate cancer and linked with tumor progression [17]. ALDH3A1 showed high expression levels under sphere-forming conditions. Yan and co-authors performed immunohistochemical staining in xenografts of prostate cancer stem cells and demonstrated the elevated expression of ALDH3A1 compared to injected monolayer cells. The ALDH3A1 expression was significantly elevated in experimental PCa lung metastases compared to subcutaneously injected DU145 monolayer cells in xenograft tumors. The clinical data suggest an increased level of ALDH3A1 in prostate cancer samples over prostate intraepithelial neoplasia and healthy prostate specimens. As for the xenograft tumor staining, elevated levels of ALDH3A1 were found in lymph node and bone metastases compared to primary tissues. Taken together, this finding indicates that ALDH3A1 is associated with tumor progression [17]. In contrast, Le Magnen and colleagues revealed a decreased expression of ALDH3A1 in prostate cancer cells over benign prostate hyperplasia. However, both sample sets displayed higher ALDH3A1 expression in malignant specimens than in the healthy prostate tissues [49].

The induction of Pim1 or c-Myc oncogenes’ expression in RWPE1 cells both led to a decrease in ALDH3A1 levels. Although induced Pim1 expression did not lead to a malignant transformation of RWPE1 cells, the authors showed increased tumorigenesis in LNCaP and DU145 cells. [112]. On the other hand, the induced upregulation of a tumor suppressor protein Latexin also led to a significant decrease in ALDH3A1 expression in primary non-malignant prostate epithelial cells. Latexin-related signaling mechanisms might represent a way in which ALDH isoforms’ expression is mutually regulated as all-trans retinoic acid (for which synthesis is catalyzed by ALDH1A1, ALDH1A2, ALDH1A3, and ALDH8A1) increased the expression of this protein. Further research could elucidate how exactly ALDH3A1, Latexin, and various other ALDH isoforms are related to each other during prostate cancer progression [113].

The administration of root extract of *Angelica gigas* mainly containing decursin and decursinol angelate decreased levels of ALDH3A1 in the prostate tissues of a mouse by 40%. With its proven effect on curbing adenocarcinoma development in TRAMP mice, *Angelica gigas*’ extract could present a way to target ALDH3A1 expression and simultaneously slow down cancer progression [114,115]. Another study revolving around using the previously mentioned curcumin (see section ALDH1B1) revealed its inhibitory effect on PCa metastasis development. Curcumin treatment inhibited CXCL1, CXCL2 and ALDH3A1 expression, and knockdown of CXCL1 or CXCL2 had an even more pronounced inhibitory effect on ALDH3A1 expression. Therefore, ALDH3A1 appears to be regulated by CXCL1 and CXCL2- induced signaling axes [116]. Whether ALDH3A1 is a mechanistic driver in promoting metastases or instead acts as a sole marker needs to be revealed by further investigation. 

ALDH3A1 is crucial in overcoming chemoresistance, while treatment with 5,8-dihydroxy-2-[(1R)-1-hydroxy-4-methyl-pent-3-enyl]naphthalene-1,4-dione (shikonin)—a substance present in the roots of *Lithospermum erythrorhizon*—decreased ALDH3A1 expression through the inhibition of ABCG2. The authors demonstrated that inhibiting ALDH3A1 by CB29 (an inhibitor that specifically targets ALDH3A1 but not ALDH1A1, ALDH1A2, ALDH1A3, ALDH1B1, or ALDH2) or treatment with shikonin converts DU145 cells with acquired cabazitaxel (a chemotherapeutic drug used for patients with CRPC) resistance to a cabazitaxel-sensitive state. This study demonstrated a link between ABCG2 and ALDH3A1 in promoting chemoresistance in PCa and proposed them as targets for a combined therapy [117,118].

A tissue microarray study analyzing the expression of multiple markers for castration-resistant stem cells in two independent datasets (one on protein and one on the mRNA level) revealed that ALDH3A1 significantly and positively correlated with the expression of SOX2 and NANOG in hormone-naïve patients but negatively correlated with NKX3.1 in castration-resistant specimens. NKX3.1 is a marker of castration-resistant prostate stem cells but also drives PCa differentiation to luminal phenotype [87]. NKX3.1 is an androgen-regulated gene, whereas NANOG co-occupies and represses many AR transcription loci involved in differentiation, including NKX3.1, disregarding the presence of androgen and therefore reprograms PCa to a castration-resistant and stem cell-like state [119]. As ALDH3A1 expression correlates with NANOG and is associated with increasing PCa aggressiveness, ALDH3A1 can also be important in PCa progression to an androgen-refractory state. The ALDH3A1 correlation with both NKX3.1 and NANOG hints at an interconnection with the AR; however, their potential relation is still debatable. 

ALDH3A1 was upregulated in response to the transient activation of the MAPK pathway by 17β-estradiol in prostate progenitor cells [120]. In a study focusing on the culturing of PCa cells under solid, soft, and fluid conditions, ALDH3A1 expression was elevated upon culture in suspension. This effect also persisted during long-term suspension culture and was reversible upon the re-introduction of cells in a monolayer culture. This observation does not only further reinforces the ALDH3A1 status as a potential marker for prostate CSCs but hints at its possible role in tumor dissemination and combating shear stress. Furthermore, the plasticity of the ALDH3A1 expression may potentially explain why Federer-Gsponer and co-authors found a limited number of PCa specimens expressing high amounts of ALDH3A1 [87,121].

## 10. ALDH3A2

Generally, there are diverging results regarding trends in the expression of ALDH3A2 during PCa progression. A study conducted with patients’ samples collected at the Karolinska Institute Sweden and the John Hopkins Hospital showed that its expression decreased in primary cancerous tissues compared to the healthy prostate specimens. A further reduction was observed when compared to the metastatic tissues [31,106,122]. Van den Hoogen and co-workers suggested that ALDH3A2 does not contribute to the ALDH activity as detected by the ALDEFLUOR assay as no immunohistochemical staining was found in the primary tumor or normal prostate specimens, despite detecting high levels of ALDH3A2 mRNA in both the PCa cell lines and primary culture samples [37]. On the other hand, a study employing an established LNCaP cell line, its derivatives, and corresponding xenograft models showed that high expression of ALDH3A2 isoform could potentially be associated with an increased PCa aggressiveness and CRPC development. In particular, Ferrari et al. demonstrated that ALDH3A2 was expressed higher in cells cultured in the presence of dihydrotestosterone together with antiandrogen bicalutamide as compared to androgen-independent cells cultured with bicalutamide alone, suggesting that ALDH3A2 might play a role in the development of the intermediate cellular phenotype during PCa progression to an androgen-refractory state [123]. Furthermore, enzalutamide, apalutamide, and bicalutamide treatment led to increased ALDH3A2 expression in androgen-dependent LNCaP cells [124]. However, cells with acquired enzalutamide resistance expressed lower levels of ALDH3A2 compared to parental LNCaP cells. Possibly, the role of ALDH3A2 transiently changes during PCa progression and after ADT [125]. 

Other studies have focused on the effects of cytotoxic drug docetaxel on the gene expression profile and activity of metabolic genes. It was shown that ALDH3A2 expression did not change upon docetaxel treatment, but its higher metabolic activity was observed in PC3 cells. It might be attributed to the aldehyde-containing products of docetaxel metabolism [126]. The treatment of C4-2B and PC-3M-luc2 cells with ethanol extracts from neem leaves (which contain a plethora of triterpenoids, non-terpenoids, meliacins, phenolics, limonoids, and flavonoids) decreased the proliferation in vitro and tumor growth in vivo and resulted in elevated ALDH3A2 transcript levels [127,128,129]. Altogether, ALDH3A2 represents a very intriguing isoform since its levels are downregulated in cancerous tissues but increased in response to various treatment approaches. These findings potentially hint at a tumor suppressor role of ALDH3A2.

## 11. ALDH3B1

Limited information is available about ALDH3B1 in prostate cancer. It was detected in different experimental conditions as part of various signatures containing other ALDH isoforms described in more details in the sections ALDH7A1 and ALDH16A1 [125,130].

## 12. ALDH3B2

Wu and colleagues acquired data about differentially expressed genes between CRPC and non-CRPC. They employed a search for protein–protein interactions among the genes with an altered gene expression and found ALDH1A3 and ALDH3B2 as genes with high connectivity. While ALDH3B2 was not shown as one of the hub genes in the development of CRPC, it may still have significance for cancer progression as specific ALDH3B2 variants were correlated with the risk for colorectal cancer (CRC) [131,132]. ALDH3B2 was upregulated in PCa cells co-cultured with cancer-associated fibroblasts. ALDH3B2 is known to be a target of miR-143-5p, and an analysis of differential expression of microRNAs (miRNAs) and their potential targets in a publicly available PCa microarray datasets found miR-143-5p, a potential tumor suppressor, to be downregulated with a subsequent ALDH3B2 upregulation [133,134]. Finally, induced c-Myc or Pim1 expression in RWPE1 cells decreased the expression of ALDH3B2, uncovering a potential mechanism of its regulation [112].

## 13. ALDH4A1

ALDH4A1 expression was positively and directly regulated by p53 [135,136]. ALDH4A1 was connected to ALDH7A1 as a stable ALDH7A1 knockdown resulted in increased ALDH4A1 levels in PC-3M-Pro4lucA6 cells compared to the untransfected counterparts [38]. ALDH4A1 expression was shown to be directly regulated by androgens [137], and the stimulation of DUCaP cells with the synthetic androgen R1881 resulted in the binding of the AR to an androgen receptor binding site close to the ALDH4A1 gene and upregulated its expression [138]. The miR-1290 encoding sequence is located in the ALDH4A1 gene locus. Highly expressed miR-1290 was associated with the activation of the WNT pathway and increased the levels of c-Myc and NANOG in colon cancer. It would be of great interest to investigate a potential connection of AR with both ALDH4A1 and miR-1290 and their downstream regulations in prostate cancer [138,139]. ALDH4A1 was upregulated in response to the treatment with an inhibitor of androgen synthesis 3-keto-5α-abiraterone and monoamine oxidase A inhibitor clorgyline. The simultaneously increased AR transcript levels suggest a potential explanation of the ALDH4A1 upregulation [140,141].

ALDH4A1 was also linked to proline degradation, and patients with a lower expression of proline metabolism genes displayed a lower biochemical recurrence-free survival. Another ALDH isoform, ALDH18A1, catalyzes the reactions towards proline synthesis. Therefore, the equilibrium between both isoforms can be of great interest for understanding the importance of proline cata- and anabolism in prostate cancer [34].

## 14. ALDH5A1

Two studies by Ippolito and colleagues demonstrated the importance of a metabolic pathway involving the synthesis of γ-aminobutyric acid (GABA) by glutamate decarboxylase (GAD1). ALDH5A1 catalyzes the reaction of succinic semialdehyde to succinate and is part of the GABA utilization in cellular energy metabolism. GABA synthesis was connected to the pathogenicity of metastatic prostate neuroendocrine cancers. The study showed that glutamine deprivation in PCa might improve cancer therapy as GABA synthesis and oxidizing pathways are decreased. The authors demonstrated a trend for decreased ALDH5A1 activity under glutamine but not glucose deprivation, and stable activity of the ALDH5A1 enzyme was observed under acidic and alkaline conditions. The authors concluded that ALDH5A1 activity might depend on post-translational modifications, and that glutamine deprivation can be a promising treatment for anticancer therapy. Taking into account that the reaction catalyzed by ALDH5A1 yields a reduced form of nicotinamide adenine dinucleotide (NADH), it was suggested that GABA serves as an energy deposit that can be reintroduced into the tricarboxylic acid (TCA) cycle by ALDH5A1 to generate a reducing equivalent for oxidative phosphorylation [35,142]. In a study investigating the function of PSA in PCa, Li et al. discovered ALDH5A1 to be upregulated upon a PSA knockdown in C4-2 cells [130].

## 15. ALDH6A1

As for ALDH4A1, the ALDH6A1 isoform appears to be interrelated with ALDH7A1 since its knockdown led to a minor increase of the ALDH6A1 mRNA level [38]. Cho et al. suggested ALDH6A1 as part of a three-gene signature including Hsp27 and prohibitin associated with survival of patients with metastatic prostate cancer. The authors also investigated the expression levels of ALDH6A1 in normal, cancerous, and metastatic prostate samples. ALDH6A1 expression was increased in tumor samples and even further increased in metastatic PCa tissues. However, within the metastatic samples, a high ALDH6A1 expression was confined to lymph node and nerve metastases, while bone metastases showed low levels of ALDH6A1 [143]. Two studies showed that ALDH6A1 exhibited a low expression across many cancers, but contrarily demonstrated its increased expression in PCa compared to healthy prostate specimens or BPH [25,36]. Such a specific upregulation of ALDH6A1 in PCa tissues may be potentially explained by its dependence on androgen signaling and by the importance of the AR in the progression of PCa since the treatment of LNCaP cells with antiandrogen bicalutamide decreased the ALDH6A1 protein level [144]. Interestingly, ALDH1A3 and ALDH6A1 expression was downregulated in NEPC [145]. This indicates that ALDH6A1 may play different roles in the onset and progression of NEPC and conventional acinar adenocarcinoma.

## 16. ALDH7A1

ALDH7A1 is one of the most important and well-investigated isoforms for prostate cancer and is considered a stemness marker [37,38]. Van der Hoogen and colleagues closely explored the importance of ALDH7A1 and found elevated transcript levels in PCa cell lines and primary tissues [37]. Similarly, Le Magnen et al. observed higher ALDH7A1 levels in clinical PCa samples over BPH or a normal prostate [49]. Immunohistochemical staining demonstrated that ALDH7A1 was highly expressed in primary tumor and bone metastatic samples [37]. In an Affymetrix gene expression study performed on 24 androgen deprivation-resistant metastatic samples (of four patients) and primary PCa tissues, lower ALDH7A1 expression levels were found in the pooled metastatic samples from lymph nodes, lung, liver, and adrenal gland of each patient compared to the primary tissue expression [146]. Importantly, bone metastatic tumor samples were not included in this study.

Van den Hoogen and co-authors conducted a follow-up study solely interested in ALDH7A1. The stable ALDH7A1 knockout in the PC3 cell line resulted in decreased clonogenicity and migration while not affecting the proliferative capacity. The knockdown concomitantly decreased several stemness and pro-metastatic markers. The downregulated transcription targets included transcriptional repressors of E-cadherin: SNAIL, SNAIL2, and TWIST. As a result, the E-cadherin to vimentin ratio significantly increased, hinting at a rather epithelial and less invasive phenotype. In an in vivo mice model, fewer tumor nodules, bone metastases, and tumor burden were observed upon the ALDH7A1 knockdown. The authors also showed that TGF-β induces the expression of ALDH7A1 and increases the ALDH^+^ population, while BMP2, BMP4, and BMP7 decreased both the ALDH7A1 expression levels and ALDH^+^ population. These findings indicate the immense importance of this isoform for the generation of bone metastases in PCa and suggest a reason for the delayed metastatic spread to the bones upon ALDH7A1 knockdown by decreasing osteopontin and CD44 expression [38]. Similarly, Chen and colleagues demonstrated the importance of ALDH7A1 in developing the metastases in the caudal hematopoietic tissues (CHT) of zebrafish. Six days post-injection of the bone metastatic cell line PC-3M-Pro4, they found an elevated expression of various CSC markers, including ALDH7A1, compared to cells cultured in vitro [147].

A signature of ALDH3B1, ALDH4A1, ALDH6A1, and ALDH7A1 was downregulated upon overexpression of the orphan nuclear receptor estrogen-related receptor β (ERRβ), one of the regulators of reprogramming towards induced pluripotent stem cells (iPSCs), and cell treatment with its ligand DY131 [148,149]. Like ALDH5A1, ALDH7A1 was upregulated in response to the PSA knockdown [130]. An RNAseq revealed a robust expression of this isoform in about 60% of 77 circulating tumor cells (CTC) isolated from 13 patients [150]. Another study proved an important role of the WNT/β-catenin signaling pathway in acquiring resistance against enzalutamide, whereas the transcript levels of ALDH7A1 were upregulated in an enzalutamide-resistant cell line compared to its non-resistant predecessor [70]. Teschendorff and colleagues investigated the signaling entropy of CTCs’ transcriptomes to measure their differentiation potential. They employed a dataset of 73 CTCs that were isolated from 11 patients with CRPC, of which five displayed disease progression under the enzalutamide treatment. CTCs of patients harboring enzalutamide resistance displayed a higher signaling entropy indicative of a higher differentiation potential. The expression correlation of various CSC markers with the signaling entropy revealed ALDH7A1 as indicative of enzalutamide-resistant prostate CSCs [151]. This isoform is also a part of a panel of upregulated transcripts in PCa used to predict for a worse disease-free survival. The higher expression of the panel genes is associated with metabolic changes in PCa, particularly an increased utilization of pyruvate and succinate for energy production [152]. A proteomic analysis by Kwon and co-authors revealed an upregulation of ALDH7A1 in both the androgen-independent (PC-3M) and androgen-dependent (LNCaP-LN3) cell lines compared to their non-aggressive counterparts (PC3 and LNCaP, respectively) [153]. Similarly, Chen and colleagues serially passaged LAPC9 cells as xenografts in castrated mice modeling androgen independence and intact mice for androgen dependence. In this study, ALDH7A1 expression was elevated in AR-independent xenograft tumors. Both studies indicate an important role of ALDH7A1 in PCa progression [154].

Furthermore, ALDH7A1 was also upregulated under an acquired resistance to zoledronic acid (an inhibitor of osteoclast bone resorption) in PCa cell lines [155]. The elevated expression was reversible upon the knockdown of annexin A1 (ANXA1), a protein shown to an orchestrate invasion and tied to the expression of the matrix metalloproteinases (MMPs) and focal adhesion kinase as well as facilitating EMT. Thus, ALDH7A1 emerges as a mediator of the ANXA1-related signaling, and both could be essential targets for the attenuation of PCa aggressiveness [156].

In response to treatment with imipridone ONC201—a small molecule inhibitor that exerts anti-CSC effect by inhibiting Akt/ERK and activating the DR5/TRAIL pathways—multiple CSC markers including ALDH1A1 and ALDH7A1 were downregulated in the DU145 cells [157]. The PCa cell line with induced pluripotency iPS87 displayed tumor-initiating capabilities and stem cell-like features and has been proposed as a model to test CSC-targeting therapies in PCa. The cell line possesses an upregulated expression of ALDH7A1 and other stemness markers, including transcriptional factors SOX2, OCT4, and NANOG. Of note, ALDH7A1 was found to be localized in the nucleus and cytoplasm, although nuclear functions on ALDH7A1 warrant further investigation [158]. The same group conducted a follow-up study using this cell line in the context of fibroblast growth factor receptor (FGFR) signaling in PCa that has been associated with advanced disease. The authors used various tyrosine kinase inhibitors (PD166866, BGJ398, and dovitinib) to inhibit FGFR signaling. ALDH7A1 expression was upregulated in DU145, PC3, and iPS87 spheroids in response to dovitinib treatment, suggesting its potential role in activating the pro-survival mechanisms [159].

PCa progression is associated with an increased nuclear localization of the ALDH7A1 protein. In contrast to cancer cells, ALDH7A1 has a cytosolic localization in BPH samples. [160]. Altogether, ALDH7A1 has been proposed as a marker for PCa and stemness involved in the formation of bone metastases. A computational analysis has shown that high ALDH7A1 expression in CTCs can predict for enzalutamide resistance of PCa. Inhibition of ALDH7A1 decreased the expression of other stemness and metastatic markers and represents a promising target to treat bone metastases and increase the enzalutamide sensitivity in PCa.

## 17. ALDH8A1

To the best of our knowledge, only little PCa-related research has been conducted on this retinal converting isoform. ALDH8A1 was detected as part of a multiple ALDH-gene signature, where it was suggested to be transcriptionally regulated by the aryl hydrocarbon receptor (AHR) nuclear translocator complex [125].

## 18. ALDH9A1

ALDH9A1, along with ALDH5A1, promotes the dehydrogenation of gamma-aminobutyraldehyde (ABAL) to GABA. Bova and colleagues discovered a p.W89R missense mutation of ALDH9A1 in a whole-genome and transcriptome sequencing study of a multiple PCa metastases of a single patient. They validated it on the transcript level and showed its presence in all analyzed metastases from the different anatomical sites in a single patient [161]. Verma and colleagues compared the transcriptomic profile of LNCaP cells with an acquired enzalutamide resistance with their parental counterparts and found the downregulation of ALDH9A1 expression in the resistance cells [162]. In another comparative study, Stevenson et al. showed that ALDH1A1, ALDH2, and ALDH9A1 proteins are present in lymph node metastasis specimens of breast cancer, pancreatic ductal adenocarcinoma, and prostate cancer, indicating a potential common role of these proteins in the development of lymph node metastases in patients with different tumor entities [163]. Additionally, ALDH9A1 was shown to be associated with a biochemical recurrence in the Cancer Genome Atlas prostate adenocarcinoma (TCGA-PRAD) dataset [164].

ALDH9A1 is a predicted target of the miRNA-711, which inversely correlates with the Gleason score and metastases. While miRNA-711 potentially plays a tumor-suppressing role in PCa, its precise interaction with ALDH9A1 and the implications for tumor suppression or progression remains to be elucidated [165]. Voss and co-authors transfected DU145 cells with miRNA-96, shown to be positively associated with CRPC in 49 assessed patients, and found the mRNA of ALDH9A1 isoform among the top transcripts present in the Argonaut-2 complex, indicating its degradation [166]. Although both miRNA-711 and miRNA-96 induce the degradation of the ALDH9A1 mRNA, their different association with PCa progression could be explained by silencing additional targets other than ALDH9A1 [165,166]. Another study used the patient-derived explant (PDE) model to demonstrate that tumors poorly responding to treatment with the Hsp90 inhibitor 17-AAG possess an increased expression of ALDH9A1 and other proteins involved in metabolic pathways. Further studies may elucidate whether this isoform directly plays a role in alleviating the therapeutic effect of 17-AAG [167].

Prostatitis highly correlates with PCa incidence [168]. In the seminal fluid of men with prostatitis, ALDH9A1 expression was 2-fold decreased compared to the seminal fluid of healthy patients [169]. These findings align with a study where Aiderus and colleagues unraveled a 19-gene signature involved in fatty acid oxidation also containing ALDH9A1. This signature was significantly associated with patients’ outcomes in breast cancer. The authors validated their findings on prostate cancer and found a significantly decreased expression of this signature in metastatic tissues than in the primary tumor specimens or normal tissue samples [170].

## 19. ALDH16A1

As for today, the data on the role of ALDH16A1 isoform in PCa development is limited. Verma and colleagues employed a microarray study to compare several genes of the aryl hydrocarbon receptor (AHR) signaling pathway in TRAMP mice versus non-transgenic counterparts. The authors found the deregulated signature of the isogenes ALDH1A2, ALDH1A3, ALDH1L2, ALDH3B1, ALDH8A1, ALDH9A1, and ALDH16A1 genes, which are possible targets of transcriptional activation by the AHR nuclear translocator complex [125].

## 20. ALDH18A1

ALDH18A1 is the last member of the ALDH gene family also named delta-1-pyrroline-5-carboxylate synthase (P5CS). The c-Myc gene directly regulates the expression of ALDH18A1 to increase its protein levels [171]. Although c-Myc is one of the most frequently upregulated genes in PCa, Tian and co-authors did not find significant differences in the expression level of ALDH18A1 when comparing the TCGA-PRAD dataset to the panel of multiple TCGA datasets comprising 11 tumor entities [172].

ALDH9A1 and ALDH18A1 are upregulated in PCa CTCs compared to primary tumors [173]. ALDH18A1 expression is potentially hypoxia responsive as it was upregulated in castration-resistant LNCaP-abl cells after 24 h of treatment with the hypoxia-mimicking drug dimethyloxalylglycine. However, the hypoxia-induced ALDH18A1 expression might depend on the cell context as it was unaltered in the castration-resistant LNCaP-abl-Hof cell line. The LNCaP-abl-Hof cell line was established as a derivative of LNCaP-abl cells after in vivo and in vitro culturing [174]. 

ALDH18A1 was significantly downregulated upon the treatment of PC3 cells with curcumin. Concomitantly, isoforms ALDH1A3 and ALDHB1 were also downregulated considerably, and ALDH7A1 was upregulated. The authors showed that treatment with curcumin leads to cell death by inducing endoplasmatic reticulum stress, an unfolded protein response, and cell cycle arrest. They also demonstrated increased ROS levels and proposed it as one of the reasons for curcumin treatments’ antitumoral effect. The downregulation of the above-mentioned ALDH isoforms involved in ROS scavenging suggests their potential role in the overall beneficial effect of curcumin treatment [97,175].

ALDH18A1 was a target of S-nitrosylation in the healthy prostate epithelial cell line NPrEC. To date, this finding was not validated in the PCa cell lines. However, this observation may provide a starting point to investigate how nitric oxide synthases influence diseases of the prostate and their pathophysiology since neoplastic malignancies can result from aberrant nitric oxide production [176,177].

Similar to ALDH4A1, ALDH18A1 is also under positive transcriptional control of p53 and involved in the production of pyroline-5-carboxylate (P5C), a precursor for proline synthesis [41]. As previously mentioned, patients with a lower expression of genes involved in proline degradation displayed a shorter biochemical recurrence-free survival. Simultaneously, the expression of proline synthesis genes was increased in prostate cancer specimens. These results suggest that the maintenance or increase of proline levels is a possible PCa metabolic adaption [34]. In particular, the equilibrium of proline synthesis and degradation can be used to maintain the redox levels while regulating the NAD+/NADP+ ratio [178,179]. Furthermore, Yan and colleagues showed that P5C released by prostate cancer cells could decrease T cell proliferation and their capability to produce cytokines, thereby evading the immune response. Since both ALDH18A1 and ALDH4A1 are heavily involved in both proline and P5C synthesis and degradation, it is tempting to further interrogate the role of both isoforms in PCa development and immune evasion [180].

## 21. ALDH-Targeted Therapies for PCa Patients

The identification and isolation of ALDH^+^ cells as CSC populations is widely accepted for PCa [37,47], as well as breast [46], ovarian [181], lung [182], liver [183], stomach [184], and other solid tumors. Modern conventional therapies can eliminate most non-CSCs, while CSCs often remain radiation and drug resistant, leading to a tumor relapse and metastases. Targeting CSCs might be a powerful tool to overcome cellular resistance and increase the efficiency of the current cancer treatment strategies. ALDHs as functional markers and regulators of stemness phenotype and properties represent an attractive target for eradicating the CSC populations (Table 2).

The ALDH inhibitors can be categorized into multi-ALDH isoform inhibitors and isoform-specific inhibitors. Their inhibitory effect can also occur directly or indirectly through other signaling pathways. DEAB is a broadly used in vitro inhibitor of ALDH as it is included as a negative control in the ALDH enzymatic assays commonly employed for the detection of CSCs. Its role as an ALDH inhibitor was extensively studied for breast and ovarian cancer. DEAB reduced radiation and chemotherapy resistance of ALDH^+^CD44^+^ breast CSCs and decreased the population of CD133^+^ ovarian CSCs [190,191]. Despite its wide use as part of the ALDEFLUOR assay kit, only one study showed that using DEAB reduced sphere-forming capacities in prostate cancer cell lines and clinical human prostate samples [54]. However, the DEAB does not inhibit all ALDH isoforms, which was demonstrated for several tumor entities [13]. Yet, its inhibition of ALDH7A1 (which was previously described to be elevated in bone metastases) is of an irreversible nature [192]. The dimethyl derivative (DIMATE), α, β-acetylenic N-substituted amino thiolester, is the most potent pan-ALDH inhibitor, targeting the ALDH1 and ALDH3 subfamilies [185]. Quash and colleagues revealed the growth-inhibitory effect of the DIMATE compound by targeting ALDH3, whereas ALDH1 was less responsive to inhibition [185]. 

ALDH isoform-specific inhibitors contend the toxicity problem of multi-isoform inhibitors since ALDHs are widely expressed in normal tissues. Moreover, the specific functions and signaling pathways regulated by distinct ALDH isoforms have not been fully investigated and explained. Several ALDH isoform-specific inhibitors were discovered and described for breast and ovarian cancer models, but only a few were tested in animal models [193]. To date, the experimental data on the effects of the ALDH isoform-specific inhibitors in prostate cancer models is very limited. In particular, a study by Jiang and co-authors demonstrated that silybin, a combination of flavonolignan and flavonoid polyphenolic compounds, inhibited cell growth, migration, and invasion of ALDH^+^ cells and downregulated ALDH1A1 expression in prostate cancer cell lines and xenograft tumors in the drug-treated mice [68]. Yet, in a clinical study involving 13 prostate cancer patients treated orally with silybin none of the patients displayed a formal PSA response, but persistent disease was observed [194]. Further data showed that despite a good tolerance to the treatment, only low levels of silybin was found in prostate cancer tissues. That could warrant for a prolonged treatment course to obtain an antitumor effect. However, active processes to efflux silybin or inter-tissue and inter-organ differences in its uptake should be discussed too [194,195]. A recent study by Quattrini and co-workers employed a small library of ALDH isoform-specific inhibitors previously validated for glioblastoma multiform tumors [96]. The authors have demonstrated that 6-substituted-imidazo[1,2-a] pyridine derivatives inhibited ALDH1A1 and ALDH1A3 gene expression and the proliferation of cancer cell line PC3, normal prostate epithelial cell line PNT2-C2, and benign prostatic hyperplasia cell line BPH1 [50].

Numerous studies demonstrated that known cancer-related signaling pathway inhibitors could target the ALDH^+^ prostate cancer subpopulation. ALDH expression correlated with the expression of CD44, CD133, and transcription factor signal transducer and activator of transcription 3 (STAT3). Two studies have demonstrated that the inhibition of STAT3 signaling with galiellalactone decreased the ALDH^+^ fraction, suppressed proliferation, and induced apoptosis of ALDH^+^ cells *in vitro*. Besides, tumors treated with galiellalactone had a downregulated expression of the ALDH1A1 gene compared to the vehicle-treated controls [186,196]. The treatment of prostate cancer cells with stattic, a STAT3 small-molecule inhibitor, reduced the proportion of ALDH^+^ population in cell lines and clinical prostate cancer specimens and decreased the tumor volume and the percentage of ALDH^+^ subpopulation in patient-derived tumor xenografts [187]. A protein kinase, the mammalian target of rapamycin (mTOR), exists in two distinct signal transduction complexes, mTORC1 and mTORC2. Several preclinical studies and early stage clinical trials targeting both complexes yielded promising results [197]. In their study, La Manna and colleagues used a third-generation mTOR-inhibiting drug, rapalink-1, a dual mTORC1/mTORC2 inhibitor. The rapalink-1 treatment reduced the proportion of ALDH^+^ prostate cancer cells in patient-derived organoids and significantly delayed tumor growth in the xenograft mice models [188]. 

Treatment with multityrosine kinase inhibitor sunitinib in combination with irradiation decreased the clonogenicity of ALDH^+^ prostate cancer cells in cell lines and mouse models [189]. Various clinical trials have investigated the effect of sunitinib treatment individually or in combination with chemotherapeutics on CRPC. A phase II trial elucidated that only few patients display a PSA-response and that these events are not necessarily overlapping with other clinical parameters, such as osteoblast tracer deposition [198,199]. Yet, an effect of sunitinib on serum levels of angiogenesis factors was confirmed [198]. Slightly better PSA-response rates were found in a cohort of 36 patients that underwent sunitinib treatment after having received one or two regimens of docetaxel treatment and 11 patients had a ≥30% PSA decline compared to the baseline. While few patients also reported a decrease in pain, more than half of the patients (19 out of 36) aborted the treatment due to high toxicity. The authors suggested that less toxic and more optimized treatment can be achieved by lowering the dose and decreasing the patients’ pretreatment [200]. A larger terminated phase III trial (*n* = 873) of metastatic progressive CRPC patients that had received docetaxel treatment revealed no significant difference in the overall survival time, yet there was a better progression-free survival upon sunitinib administration over the placebo [201]. However, no PCa specimens were investigated for the expression or activity of ALDH family members. 

WNT/β-catenin signaling, a pathway involved in developing a castration-resistant disease, represents an attractive therapeutic opportunity [202]. Inhibition of the WNT/β-catenin pathway with the tankyrase inhibitor XAV939 decreased the population of ALDH^+^ prostate CSCs and downregulated the ALDH1A1 gene and protein expressions [66]. Another study demonstrated that the treatment of PCa cells with the same XAV939 compound reduced the expression of ALDH1A1 but increased the expression of the ALDH1A3 gene [23]. Incubation of PCa cells with TGF-β antagonists, BMP2, BMP4, and BMP7 significantly inhibited the population of ALDH+ cells and the mRNA expression level of ALDH7A1 [38]. 

Modern research provides a strong rationale for using gene silencing technics (e.g., small interfering RNA, microRNA, CRISPR/Cas9 editing technology) to specifically target the tumor-inducing genes [203,204]. A few studies have demonstrated the therapeutic potential of ALDH genetic silencing for therapy sensitization of prostate cancer. The siRNA-mediated knockdown of ALDH1A1 and ALDH1A3 expressions resulted in radiation therapy sensitization of prostate cancer cells [23,66]. In addition, research on breast, ovarian, and colon cancer models showed that the suppression of ALDH1A1 with specific siRNAs sensitized cells to chemotherapy [205].

The development of the anti-ALDH vaccines opens new possibilities for the prevention of PCa immune escape. Recent studies on the dendritic cell (DC)-based vaccines demonstrated that the DC pulsed by the cell lysates of ALDH^+^ CSCs inhibited primary and metastatic tumor growth in the vaccinated xenograft tumor models. As for now, the CSC–DC vaccines were successfully tested for several tumor entities, including melanoma, head and neck squamous cell carcinoma (HNSCC), and breast cancer [206,207,208]. Thus, targeting CSCs with these vaccines alone or in combination with immune checkpoint inhibitors may lead to new avenues for anticancer immunotherapy.

Combination therapies are becoming routine in the development of treatment plans for cancer patients. Combining ALDH-targeted drugs with conventional therapies (e.g., radiation and chemotherapy) provides more possibilities for the preclinical testing and guides the next round of therapeutics. Along this line, Li and co-authors used the combination treatment with nanoparticles loaded with low-dose decitabine (DAC) (NPDAC) and nanoparticles with doxorubicin (NPDOX) and demonstrated a reduced proportion of CSCs with high ALDH activity in breast cancer mammospheres [209]. Another study showed that the combination of chemotherapeutic agent paclitaxel and pan-ALDH-specific inhibitor disulfiram synergistically inhibits endometrial cancer cell progression [210]. A combination treatment of gossypol, ALDH inhibitor, and phenformin (a biguanide compound) synergized ATP depletion and induced cell death in non-small-cell lung cancer cells [211]. A triple combination therapy of the chemotherapeutic drug temozolomide with gossypol and phenformin significantly reduced the ATP levels, cell viability, stemness, and invasiveness compared to temozolomide monotherapy, gossypol, and phenformin dual therapy in glioblastoma [212]. Applying the pan-ALDH inhibitor DIMATE in combination with cisplatin strongly reduced the tumor volume in a non-small-cell lung cancer model [213]. As of today, there is no information on how the combination therapy would influence prostate cancer progression. However, based on the data obtained on other tumor models, we could speculate that drug combinations of ALDH-targeted agents with chemotherapies might enhance therapeutic response in PCa, including CSC populations responsible for the therapeutic tolerance, invasiveness of therapy-surviving cells, and relapse.

## 22. Conclusions

The number of papers reporting the role of ALDHs in tumorigenesis and therapy resistance has grown vastly during the last decade. ALDH activity measured by ALDEFLUOR assay is widely used for the identification and isolation of CSCs. At present, there are many efforts to understand the role of specific ALDH isoforms in cancer progression and characterize different ALDH isoforms as potential tumor biomarkers. Accordingly, ALDH inhibitors represent promising and practical tools for the targeting and eliminating of the CSC populations. However, ALDH inhibitors with a broad specificity are potentially associated with high toxicity as ALDH proteins are widely expressed in normal tissues and stem cells, including hematopoietic progenitor cells, mesenchymal stem cells, endothelial progenitor cells, neural stem cells, myoblast progenitor cells etc. ALDH activity has also been described as a marker of normal prostate progenitor cells [205]. Although numerous reports showed the specific upregulation of distinct ALDH isozymes in PCa tissues, more research is needed to validate those findings on mouse models and clinical samples. On the other hand, ALDH activity is not highly specific for stem cell populations. Its combination with additional markers, such as CD44, α2β1, CD133 etc., is needed to more accurately analyze the stem cells in prostate tissues. While several studies demonstrated that cancer-related signaling pathway inhibitors could target ALDH^+^ cells, isoform-specific drugs are essential for efficiently targeting prostate CSC populations. Many compounds lack preclinical and clinical validation, as well as better a characterization of pharmacokinetic properties. Increasing the sensitivity and optimizing the specificity of drug delivery would improve the bioavailability and minimize the toxicity. The current clinical trials suggest that some drugs inhibiting the fraction of ALDH+ cells, such as sunitinib, displayed activity in patients with CRPC. However, these therapies are associated with toxic effects and need further optimization [200,201]. 

An additional approach to increase the efficiency of anticancer therapy targeting ALDH+ populations could be a combination treatment approach. A combination of ALDH inhibitors with conventional therapies could help to overcome the resistance without ablating normal stem cell functions. Several recent studies provided evidence that some ALDH isoforms might contribute to the tumor immune surveillance. In particular, ALDH1A3 expression in melanoma cells was correlated with programmed death-ligand 1 (PD-L1) expression in non-small-cell lung carcinoma (NSCLC) tumor samples and led to the reduced proliferation of the peripheral blood mononuclear cells in the co-culture experiments. ALDH2 was shown to upregulate PD-L1 expression in CRC cells in vitro and in tumor tissues. A low expression level of ALDH2 in CRC specimens was associated with a higher number of the tumor infiltrated CD3+ and CD8+ activated T cells. In addition, it has been shown that crosstalk between immune cells and ALDH+ tumor cells is reciprocal, as the targeting of tumor-associated myeloid cells by the inhibition of colony-stimulating factor 1 receptor (CSF1R) resulted in the depletion of the ALDH+ CSC population in pancreatic ductal adenocarcinoma (PDAC) tumors [214,215,216]. The development of the anti-ALDH vaccines and their clinical testing in combination with immune checkpoint inhibitors can be an efficient strategy to eradicate CSC populations and improve the tumor therapy response [206,207,208]. 

Finally, unraveling the functional roles of specific ALDH isoforms will facilitate the design of novel strategies for cancer therapies. In this respect, small interfering RNA or microRNA, clustered regularly interspaced short palindromic repeats (CRISPR) editing, as well as isoform-specific small molecules, will help us to determine the importance of individual ALDH genes. Employing nanoliposomal delivery [217] and osmotic minipumps for drug delivery [218] and developing PCa-specific antibody-drug conjugates to deliver ALDH inhibitors can potentially advance the ALDH-targeted therapy [219] and make it more specific for tumor tissues. 

## Figures and Tables

**Table 1:** Characteristics of the ALDH family genes. Cellular localization and notable substrates have been adapted from Kopakka and colleagues [20] and additional sources. Expression values in the TCGA-PRAD dataset are given as fragments per kilobase of transcript per million mapped reads, FPKM (standard deviation). Isoforms with an expression of less than 16 FPKM in 90% of patients were filtered, and FPKM values were confidence interval corrected across all of the patients. ^1^: affinity for 9-cis-retinal, ^2^: affinity for all-trans-retinal, N/A: information is not available.

Gene	Chr. Location	Prominent Substrate	Metabolic Pathways	Prognostic Value for PCa	Cellular Localization	Expression in TCGA-PRAD	References
ALDH1A1	9q21.13	Retinal ^1^	retinoic acid signaling	worse biochemical recurrence	cytosolic	2276.1 (5877.8)	[20,21,22]
ALDH1A2	15q21.3	Retinal ^2^	retinoic acid signaling	worse recurrence-free survival	cytosolic	367.1 (284.3)	[16,20,22]
ALDH1A3	15q26.3	Retinal ^2^	retinoic acid signaling	worse progression-free and biochemical recurrence-free survival	cytosolic	16,116.6 (10,305.5)	[20,22,23,24]
ALDH1B1	9p13.1	Retinal, acetaldehyde	ethanol metabolism; retinoic acid signaling	expression increased in PCa	mitochondrial	1430.6 (626.2)	[20,25,26,27]
ALDH1L1	3q21.3	10-formyltetrahydrofolate	tetrahydrofolate synthesis	N/A	cytosolic	496.8 (406.5)	[20,28]
ALDH1L2	12q23.3	10-formyltetrahydrofolate	tetrahydrofolate synthesis	N/A	mitochondrial	71.0 (60.7)	[20,28]
ALDH2	12q24.12	acetaldehyde	ethanol metabolism	worse recurrence-free survival	mitochondrial	2907.5 (1812.9)	[20,29]
ALDH3A1	17p11.2	aromatic and medium-chain (un-)saturated aldehydes	N/A	associated with PCa progression	cytosolic, nuclear	22.2 (27.4)	[17,20,30]
ALDH3A2	17p11.2	long-chain aliphatic aldehydes	tryptophan metabolism; fatty acid metabolism	downregulated in PCa specimens	endoplasmic reticulum	3221.9 (1277.9)	[20,31,32]
ALDH3B1	11q13.2	long-chain fatty aldehydes, octanal	N/A	N/A	endoplasmic reticulum	111.7 (79.2)	[20,33]
ALDH3B2	11q13.2	N/A	N/A	N/A	endoplasmic reticulum	914.0 (1047.1)	[20]
ALDH4A1	1p36.13	pyrroline-5-carboxylate	proline degradation	N/A	mitochondrial	2309.1 (1268.0)	[20,34]
ALDH5A1	6p22.3	succinic semialdehyde	γ-aminobutyric acid (GABA) degradation	N/A	mitochondrial	761.7 (255.9)	[20,35]
ALDH6A1	14q24.3	malonate; methylmalonate semialdehyde	valine and pyrimidine catabolism	expression increases with progression	mitochondrial	1153.5 (645.4)	[20,25,36]
ALDH7A1	5q23.2	α-aminoadipic semialdehyde	ethanol metabolism; lipid peroxidation; lysine catabolism	Increased in PCa; specifically bone metastases	cytosolic	2577.5 (1087.5)	[20,37,38,39]
ALDH8A1	6q23.3	2-aminomuconate semialdehyde; 9-cis-retinal	tryptophan catabolismretinoic acid signaling	N/A	cytosolic	expression levels did not pass the threshold	[20,40]
ALDH9A1	1q24.1	γ-aminobutyraldehyde	GABA synthesis	N/A	cytosolic	3185.3 (874.8)	[20,35]
ALDH16A1	19q13.33	N/A	N/A	N/A	transmembrane protein	588.8 (240.9)	[20]
ALDH18A1	10q24.1	glutamate	synthesis of proline, arginine, and ornithine	N/A	mitochondrial	2563.1 (736.9)	[20,41]

**Table 2:** Compounds targeting ALDH in prostate cancer.

Agent	Target	In Vitro	In Vivo	Ref.
Compounds directly targeting ALDH				
DEAB	ALDH1	Inhibited ALDH activity and sphere-forming features in prostate cancer cell lines and clinical human prostate specimens	N/A	[54]
DIMATE	ALDH1ALDH3	Suppressed ALDH activity, growth inhibition, and expression of ALDH1 and ALDH3 subfamilies	N/A	[185]
Silybin	ALDH1A1	Downregulated ALDH1A1 expression, inhibited cell migration, invasion, and growth of ALDH+ cells	Tumors from drug-treated mice had lower expression of ALDH1A1	[68]
6-Substituted-imidazo[1,2-a] pyridine derivatives	ALDH1A1ALDH1A3	Inhibited ALDH1A1 and ALDH1A3 gene expression, and proliferation in normal prostate epithelial cell line PNT2-C2, benign prostatic hyperplasia cell line BPH1, and cancer cell line PC3	N/A	[50]
Compounds indirectly targeting ALDH				
Galiellalactone	STAT3	Decreased the proportion of ALDH+ cells, inhibited proliferation, and induced apoptosis of ALDH^+^ cells	Mice with DU145 xenografts treated with drug had decreased expression of ALDH1A1 gene	[186]
Stattic	STAT3	Reduced the proportion of ALDH^+^ population in cell lines and clinical prostate cancer samples	Decreased the tumor volume in patient-derived tumor xenografts and the percentage of ALDH+ subpopulation	[187]
Rapalink-1	mTORC1/mTORC2	Decreased fraction of ALDH^+^ cells in patient-derived xenografts organoids	Enriched in ALDH+ cells in LAPC9 patient-derived xenograft model	[188]
Sunitinib	Tyrosine kinases	Combination of drug with radiotherapy decreased clonogenicity of ALDH^+^ prostate cancer cells	Inhibited the fraction of ALDH+ cells in tumors from mice	[189]
XAV939	WNT (tankyrase and β-catenin)	Reduced ALDH^+^ population	N/A	[66]
XAV939	WNT (tankyrase and β-catenin)	Decreased the expression of ALDH1A1 and increased expression of ALDH1A3 gene	N/A	[23]
BMP2, BMP4, and BMP7	Antagonist of TGFβ	Decreased the size of the ALDH^+^ population, inhibited ALDH7A1 expression in PC-3M-Pro4luc cells	N/A	[38]
